# Formal modeling and analysis of the hexosamine biosynthetic pathway: role of O-linked N-acetylglucosamine transferase in oncogenesis and cancer progression

**DOI:** 10.7717/peerj.2348

**Published:** 2016-09-27

**Authors:** Muhammad Tariq Saeed, Jamil Ahmad, Shahzina Kanwal, Andreana N. Holowatyj, Iftikhar A. Sheikh, Rehan Zafar Paracha, Aamir Shafi, Amnah Siddiqa, Zurah Bibi, Mukaram Khan, Amjad Ali

**Affiliations:** 1 Research Centre for Modeling and Simulation (RCMS), National University of Sciences and Technology (NUST), Islamabad, Pakistan; 2 School of Computer Science and IT, Stratford University, VA, United States; 3 Guangzhou Institutes of Biomedicine and Health, Chinese Academy of Sciences, Guangzhou, China; 4 Department of Oncology, Wayne State University School of Medicine and Barbara Ann Karmanos Cancer Institute, Detroit, MI, United States; 5 School of Electrical Engineering and Computer Science (SEECS), National University of Sciences and Technology (NUST), Islamabad, Pakistan; 6 College of Computer Science and Information Technology, University of Dammam, Al Khobar, Kingdom of Saudi Arabia; 7 Atta-ur-Rehman School of Applied Bio-science (ASAB), National University of Sciences and Technology (NUST), Islamabad, Pakistan

**Keywords:** Biological regulatory networks (BRNs), René Thomas, Qualitative modeling, Model checking, Cancer, Hexosamine biosynthetic pathway, O-GlcNAcylation, OGT, SMBioNet

## Abstract

The alteration of glucose metabolism, through increased uptake of glucose and glutamine addiction, is essential to cancer cell growth and invasion. Increased flux of glucose through the Hexosamine Biosynthetic Pathway (HBP) drives increased cellular O-GlcNAcylation (hyper-O-GlcNAcylation) and contributes to cancer progression by regulating key oncogenes. However, the association between hyper-O-GlcNAcylation and activation of these oncogenes remains poorly characterized. Here, we implement a qualitative modeling framework to analyze the role of the Biological Regulatory Network in HBP activation and its potential effects on key oncogenes. Experimental observations are encoded in a temporal language format and model checking is applied to infer the model parameters and qualitative model construction. Using this model, we discover step-wise genetic alterations that promote cancer development and invasion due to an increase in glycolytic flux, and reveal critical trajectories involved in cancer progression. We compute delay constraints to reveal important associations between the production and degradation rates of proteins. O-linked N-acetylglucosamine transferase (OGT), an enzyme used for addition of O-GlcNAc during O-GlcNAcylation, is identified as a key regulator to promote oncogenesis in a feedback mechanism through the stabilization of c-Myc. Silencing of the OGT and c-Myc loop decreases glycolytic flux and leads to programmed cell death. Results of network analyses also identify a significant cycle that highlights the role of p53-Mdm2 circuit oscillations in cancer recovery and homeostasis. Together, our findings suggest that the OGT and c-Myc feedback loop is critical in tumor progression, and targeting these mediators may provide a mechanism-based therapeutic approach to regulate hyper-O-GlcNAcylation in human cancer.

## Introduction

Cancer, a diverse group of diseases caused by an accumulation of genetic alterations that leads to abnormal cellular growth, ranks as a leading cause of death worldwide (World Health Organization, 2014). Genetic alterations result in activating oncogenes and inactivating tumor suppressor genes to regulate gene expression and support tumor progression. Oncogenes such as MYC, PI3K, and EGFR, are activated through mechanisms that include genetic translocation, amplification, DNA methylation, and histone modifications (Negrini, Gorgoulis & Halazonetis, 2010; Hanahan & Weinberg, 2011; Jones & Baylin, 2007). Repression of tumor suppressors such as p53 and PTEN is acquired through various chromatin modifications, deletions, and point mutations (Walsh & King, 2007). However, understanding the role of these aberrations in tumorigenesis is difficult as studies have shown that genomic alterations tend to be cancer-specific and drastically differ between human tumor types.

Despite the heterogeneity and complexity of these malignancies, key functions in tumor development are common. These hallmarks of cancer include: acquiring resistance toward programmed cell death (PCD), uncontrolled cell proliferation, reprogramming cellular metabolism to support chronic neoplastic proliferation, and activation of inflammatory responses to enable tumor growth (Hanahan & Weinberg, 2000; Hanahan & Weinberg, 2011). These nearly universal capabilities of cancer cells promote tumorigenesis and underlie the fundamentals of cancer biology. Thus, analyzing these network interactions and mechanisms of tumorigenesis will drive therapeutic development to selectively target these hallmark traits.

### Metabolic reprogramming and oncogenesis

**Figure 1:**
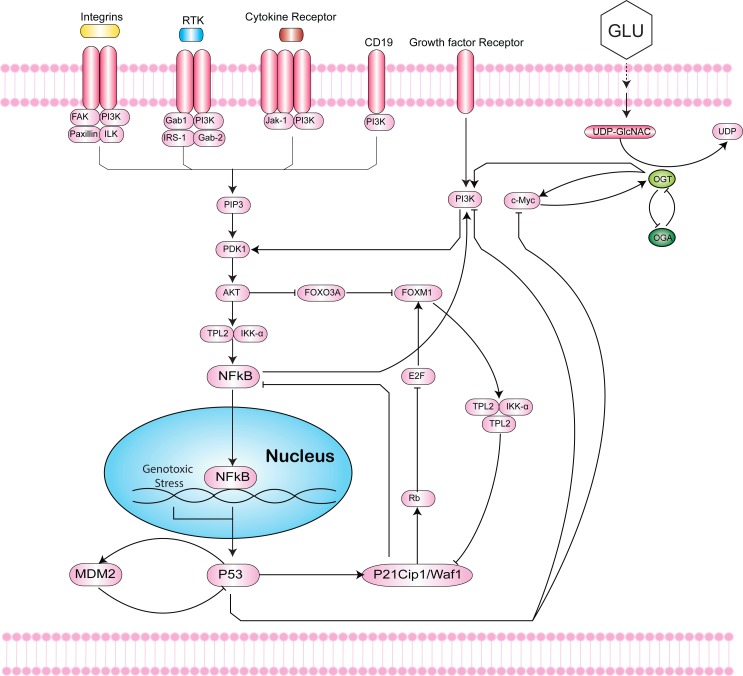
Intersection of the Hexosamine Biosynthetic Pathway (HBP), Phosphoinositide 3-kinase (PI3K)-mTOR-MYC signaling axis, and p53-MDM2 circuit. The HBP (right) generates Uridine diphosphate N-acetylglucosamine (UDP-GlcNAc) as the end product that is used by the O-GlcNAc transferase (OGT) to covalently attach O-GlcNAc to hydroxyl groups of serine/threonine residues of proteins. This dynamic process is antagonized by O-GlcNAcase (OGA). In cancer, increased HBP flux leads to hyper O-GlcNAcylation. Hyper O-GlcNAcylation of c-Myc activates the phosphoinositide 3-kinase (PI3K)-mTOR-MYC signaling axis (middle). The PI3K pathway cross-talks with Forkhead box M1 (FoxM1), an oncogenic transcription factor that is regulated by levels of O-GlcNAc and OGT (middle). Inflammatory responses to genotoxic stress induce activation of NF-*κ*B that can undergo O-GlcNAcylation to mediate genes in the immune response (left). The loss of p53 activates NF-*κ*B to increase aerobic glycolysis and support tumor metabolism. Hyper O-GlcNAcylation of p53 stabilizes the tumor suppressor and decreases p53-MDM2 interaction to block proteolysis (bottom). In response to stress, p53 can induce cyclin-dependent kinase inhibitor p21 to inhibit proliferation.

The capability of tumors to reprogram cellular metabolism and promote uncontrolled proliferation has only recently emerged as a cancer hallmark (Hanahan & Weinberg, 2011; Cairns, Harris & Mak, 2011; Dang, 2012). However, alterations of energy metabolism in cancer cells to stimulate cell growth and division first observed by Otto Warburg date back to the early twentieth century (Warburg, 1910; Weinhouse et al., 1956; Warburg, 1956). To fuel growth, tumor cells flip a metabolic switch to reprogram glucose metabolism from oxidative phosphorylation to aerobic glycolysis, and secrete lactate (“Warburg-effect”). While glycolysis produces adenosine 5′-triphosphate (ATP) faster, this process occurs at a less efficient rate. To compensate for this metabolic switch, neoplastic cells become addicted to glucose and glutamine to maintain rapid cell proliferation (Potter, 1958; Vander Heiden, Cantley & Thompson, 2009; DeBerardinis et al., 2007; Lunt & Vander Heiden, 2011; Mullen et al., 2012; DeBerardinis et al., 2008). This phenomenon of increased glycolytic flux and glucose uptake importantly increases flux into biosynthetic pathways, such as the Hexosamine Biosynthetic Pathway (HBP) (Fig. 1).

Studies have also found an association between glycolytic fueling and mutant tumor suppressors or activated oncogenes that play critical roles in evading apoptosis and promoting proliferation of tumor cells (Jang, Kim & Lee, 2013; Ying et al., 2012; Gross, Van den Heuvel & Birnbaum, 2008; Haq et al., 2013). Ying et al. (2012) found that over-expression of Kras was implicated in tumor initiation by controlling tumor metabolism and channeling glucose intermediates into the HBP. Kras upregulation of glycolytic enzymes, glucose transporters, and glutamine: fructose-6-phosphate amidotransferase 1 (GFAT1), drives increased HBP flux and cellular O-linked N-acetylglucosaminylation glycosylation (O-GlcNAcylation) in cancer (Hsu & Sabatini, 2008; Ma & Vosseller, 2013). A fundamental role of the HBP is to control O-GlcNAcylation. O-GlcNAcylation is a post-translational modification catalyzed by O-GlcNAc-transferase (OGT) that covalently adds a GlcNAc sugar moiety to hydroxyl groups of serine/threonine residues of proteins (Torres & Hart, 1984; Hart, Housley & Slawson, 2007; Issad & Kuo, 2008). This process is antagonized by O-GlcNAcase (OGA), which allows for dynamic regulation of O-GlcNAcylation in cells (Fig. 1).

Recently, O-GlcNAcylation has been proposed as a novel cancer hallmark and approach for cancer treatment due to its significant regulatory role in tumorigenesis (Fardini et al., 2013). Increased O-GlcNAcylation, termed hyper O-GlcNAcylation, and elevated OGT levels have been observed in various tumor types, including cancers of the breast, lung, liver, bladder, endometrial, prostate, pancreas, and colon (Ying et al., 2012; Gu et al., 2010; Mi et al., 2011; Zhu et al., 2012; Rozanski et al., 2012; Krześlak et al., 2012b; Lynch et al., 2012). Importantly, the inhibition of OGT has been associated with decreased proliferation of breast and prostate cancer cells (Caldwell et al., 2010; Itkonen et al., 2013). Understanding the proteins that control deregulation of cellular energy metabolism and hyper O-GlcNAcylation is needed to elucidate the mechanisms of metabolic switch in cancer cells, characterize the glycolytic phenotype, and decipher the link to cellular growth and apoptotic pathways.

### Signaling pathways

The ability of tumors to promote an inflammatory response and escape immune destruction also enables cellular proliferation and evasion of innate immunity (Dvorak, 1986; Colotta et al., 2009; Hanahan & Weinberg, 2011; Markert, Levine & Vazquez, 2012; Kroemer & Pouyssegur, 2008). Inflammation is the protective response of the innate immune system to a physiological, physical, and/or oxidative stress. The development of innate immunity is associated with the NF-*κ*B signaling cascade, where NF-*κ*B is activated through subunits of the IKK complex in response to stimuli (Karin, 2009; Hoesel & Schmid, 2013). Activation of NF-*κ*B targets and eliminates transformed cells, Disis (2010) supporting subsequent increases in apoptotic processes as an inflammatory response (Ernst, 1999; Cordon-Cardo & Prives, 1999). Further studies revealed additional roles of NF-*κ*B in controlling normal cellular and malignant processes, such as proliferation, apoptosis, and metabolism (Guttridge et al., 1999; La Rosa, Pierce & Sonenshein, 1994; Perkins, 1997; Moretti et al., 2012; Kawauchi et al., 2009; Kawauchi et al., 2008).

The subunits of NF-*κ*B contain sites for post-translational modifications to promote cross-talk with signaling pathways. O-GlcNAcylation of the c-Rel subunit of NF-*κ*B was recently demonstrated to mediate the expression of various cytokine-encoding genes involved in the immune response (Alexandrov et al., 2013). More recent studies have noted that expression of OGT, the enzyme that catalyzes O-GlcNAcylation, is correlated with c-Myc protein levels and may be involved in protein stabilization (Itkonen et al., 2013).

c-Myc belongs to the PI3K-mTOR-MYC signaling pathway, one of the most commonly mutated pathways in cancer (Fig. 1). Activation of this signaling cascade has been shown to increase hyper O-GlcNAcylation activity in breast cancer (Sodi et al., 2015). Notably, treatment of tumor cells with PI3K and mTOR inhibitors led to decreased protein expression of OGT and overall lower levels of O-GlcNAcylation. The PI3K pathway has also been reported to cross-talk with Forkhead box M1 (FoxM1), an oncogenic transcription factor (Major, Lepe & Costa, 2004). FoxM1 plays a critical role in cancer metabolism, as the reduction of O-GlcNAc levels and OGT in cancer cells is associated with a decrease in protein expression of FoxM1 in breast cancer (Caldwell et al., 2010). However, studies to date have not detected O-GlcNAc modifications on FoxM1.

In addition to NF-*κ*B and c-Myc, p53 has also been shown to be directly O-GlcNAcylated. p53 plays critical roles in DNA damage repair and apoptosis, and is one of the most frequently mutated genes in cancer. Hyper O-GlcNAcylation of p53 stabilizes the tumor suppressor and decreases p53-MDM2 interaction to block proteolysis (Fig. 1) (Yang et al., 2006). In contrast, overexpression of OGA, the antagonist to OGT, stimulates MDM2-p300 interaction and degrades p53 (Soesanto et al., 2008). Consequently, loss of p53 activates NF-*κ*B to increase aerobic glycolysis and support tumor metabolism (Kawauchi et al., 2008). Under stress, p53 can induce cyclin-dependent kinase inhibitor p21 to arrest the cell cycle and inhibit proliferation (Gartel & Tyner, 1999). Taken together, disentangling the complex interplay between NF-*κ*B, c-Myc, p53, MDM2, FoxM1, p21, and OGT is critical to understanding the roles of hyper O-GlcNAcylation, pathway signaling and cross-talk, metabolism, and programmed cell death in cancer.

### Our contribution

In this study, we examine the role of hyper O-GlcNAcylation in cancer progression by regulating the activation of oncogenes. We construct a qualitative Biological Regulatory Network (BRN) comprised of important entities involved in O-GlcNAc signaling to demonstrate activation and inhibition relationships. Model parameters are computed from known experimental observations by using a formal verification technique, called model checking. These parameters are used to translate BRN into a qualitative model which highlights important behaviors as trajectories, stable states, and cycles. Network analysis of the qualitative model is performed to identify important trajectories involved in oncogenic activation, cancer progression, and recovery. We identify significant cycles that represent normal behavior of the overall system and use hybrid modeling to compute delay constraints, which limit the system to maintain homeostasis. A similar modeling approach has been used in the past for formal modeling of biological networks, including the MAL-Associated Biological Regulatory Network (BRN) (Ahmad et al., 2012), the regulatory network of dengue virus pathogenesis and clearance (Aslam et al., 2014), the mechanism of tail resorption in tadpoles (Khalis et al., 2009), and the immunity control mechanism in bacteriophage lambda (Richard, Comet & Bernot, 2006). The results of our study highlight that O-GlcNAc transferase (OGT) plays an important regulatory role in oncogenic activation. The qualitative model reveals that persistent over-expression of OGT and c-Myc leads to deadlock state, from which the system cannot proceed to a recovery state. Another important insight obtained from the model is that silencing of the OGT and c-Myc loop decreases glycolytic flux and results in programmed cell death. Based on the results of network analysis carried out using Cytoscape, we identify a significant cycle, which highlights the important role of p53-Mdm2 oscillations to bring the system towards recovery state. The results of hybrid modeling suggest delay constraints to maintain homeostasis. We compare the important insight gained through computational modeling to show that results are in agreement with previous studies. Together, our findings suggest that the OGT and c-Myc loop is critical in tumor progression, and targeting these mediators may represent a novel therapeutic strategy to regulate hyper-O-GlcNAcylation for the treatment of cancer.

## Methods

In practice, elements of a biological system interact with each other in a positive or negative manner, that is, the expression level of an entity (gene or protein) may favor or degrade the *rate of synthesis* of other entities or itself. Usually, these systems are described using continuous modeling approaches that use a set of ordinary or partial differential equations, which are often highly non-linear, and even simple systems involving only few entities cannot be solved analytically (De Jong, 2002; Karlebach & Shamir, 2008). Secondly, differential equations involve time derivatives of quantitative data (concentration levels, reaction rates etc.), which in many cases can not be measured experimentally. These limitations paved the way towards qualitative description of biological systems with discrete variables, having limited expression levels, often only two (0 or 1). Thomas, in the 1970s, proposed a logical formalism based on qualitative representation of biological regulations (Thomas, 1973; Thomas, 1991; Thieffry & Thomas, 1995). The qualitative modeling approach, described by Thomas, employed *directed graphs* (also called *interaction graphs*) to represent the topology of a Biological Regulatory Network (BRN).

**Figure 2:**
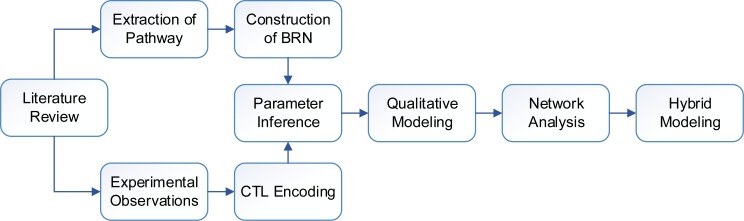
Flow diagram of the study showing sequence of methods.

The work presented in this paper mainly employs a qualitative framework for modeling biological regulations. The methods used in this study (Fig. 2) are discussed in subsequent subsections. First, we construct a qualitative Biological Regulatory Network (BRN) which is comprised of important entities isolated from signaling pathways. The unknown model parameters are inferred by encoding experimental observations into a model checker. The qualitative BRN is then translated to a stategraph by using Thomas’s framework. Important behaviors in a stategraph, such as steady states, oscillations (cycles) and important trajectories are identified. Finally, we use hybrid modeling to compute delay constraints which limit the system to remain in a normal cycle (homeostasis).

### Qualitative modeling framework

The qualitative modeling framework introduced by René Thomas uses a graph-theoratic approach to model BRNs. Each BRN is modeled as a weighted directed graph in which nodes represent biological entities such as genes or proteins, whereas the activation and inhibition relationships between nodes are represented by edges. Here, we briefly introduce semantics of the qualitative modeling framework, mainly adopted from Bernot et al. (Bernot et al., 2007; Bernot et al., 2004; Saeed & Ahmad, 2014).

**Figure 3:**
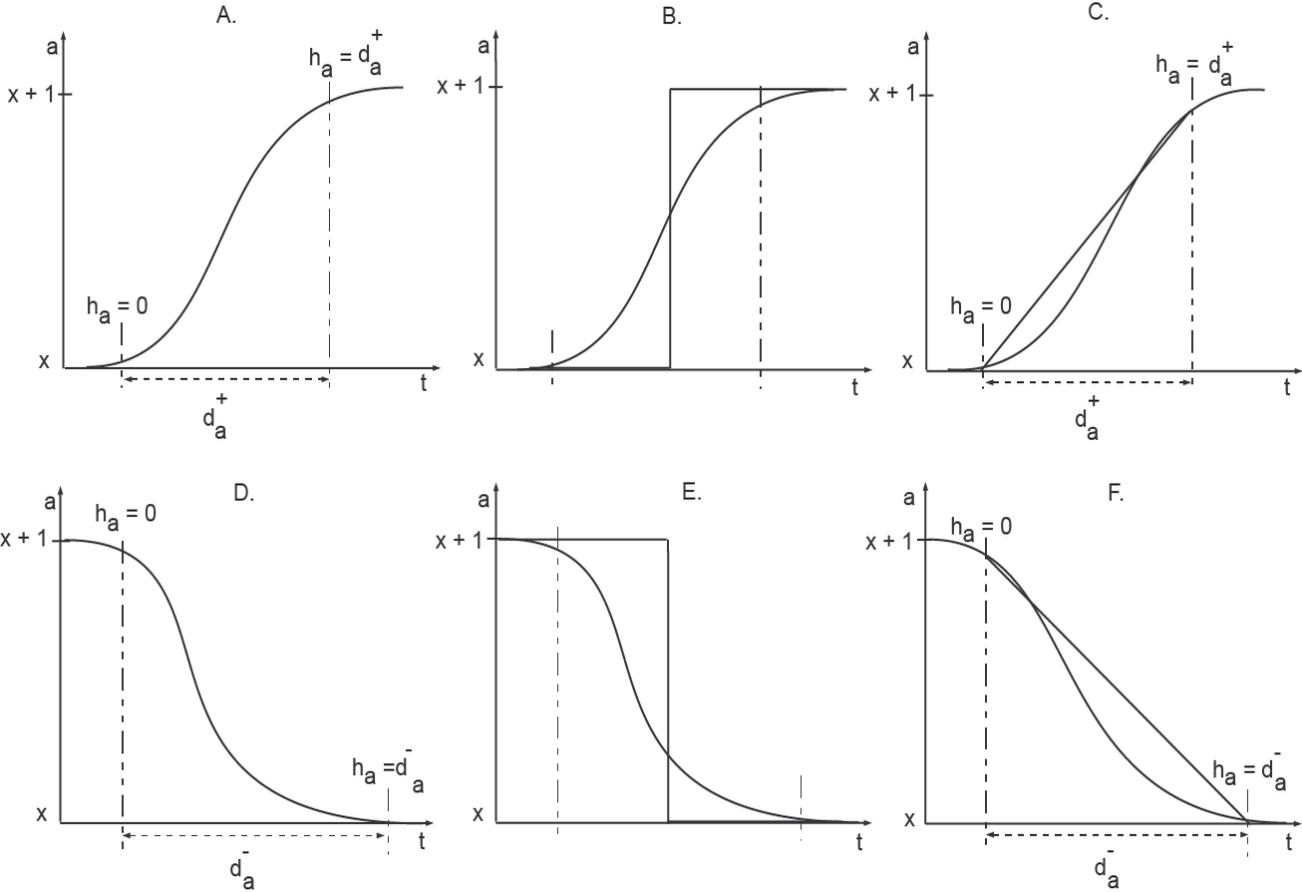
Activation and Inhibition delays (adopted from Aslam et al., 2014). The clock *h*_*a*_ measures the time of evolution between two discrete levels. Initially the clock is set to zero and the changes in the level occurs in a delay time *d*^+∕−^.

“

Definition 1 **Directed Graph**

A directed graph *G* is an ordered pair *G* = (*V*, *E*), where

• *V* is the set of all *vertices* or *nodes*
• *E* is an ordered pair of nodes i.e., if *e* ∈ *E*, then *e* = (*v*_*i*_, *v*_*j*_) and $V= \left\{ {v}_{i},{v}_{j} \right\} $.

The edge (*v*_*i*_, *v*_*j*_) is directed from *v*_*i*_ to *v*_*j*_, where *v*_*i*_ is called the head and *v*_*j*_ is called the tail. In *G*, the set of predecessors and successors of a node *v*_*j*_ are denoted as ${G}_{{v}_{j}}^{-}$ and ${G}_{{v}_{j}}^{+}$, respectively.

Definition 2 **Biological Regulatory Network**

A Biological Regulatory Network (BRN) is a labeled directed graph *G* = (*V*, *E*), where biological entities are represented by set of nodes *V* and interactions are represented by set of edges *E*⊆ *V* × *V*. Each edge (*v*_*i*_, *v*_*j*_) is labeled by a pair (*τ*, *σ*), where *τ* is the threshold at which gene *u* starts regulating gene *v*, and $\sigma = \left\{ +,- \right\} $ is called sign of interaction (+ for activation and − for inhibition).

Each node *v*_*i*_ ∈ *V* has its abstract expression level in the set *δ*_*v*_*i*__ = {0…ℓ_*v*_*i*__} where ${\ell }_{{v}_{i}}\leq \left\vert {G}_{{v}_{i}}^{+} \right\vert $. The *state* of a *BRN* is a configuration of expression levels of all entities at a particular time instant.

Definition 3 **State**

A State of BRN is *n*-tuple $S= \left\{ {s}_{{v}_{1}},..,{s}_{{v}_{n}} \right\} $, ∀*s*_*v*_*i*__ ∈ *δ*_*v*_*i*__, where *s*_*v*_*i*__ is the abstract expression level of *v*_*i*_.

In a given state, each *v*_*i*_ is regulated by its predecessors ${G}_{{v}_{i}}^{-}$, formally denoted as set of *resources*, *ω*_*v*_*i*__, defined as follows:

Definition 4 **Resources**

Let *G* = (*V*, *E*) be a BRN. The set of resources *W*_*v*_*j*__ at level *s*_*v*_*j*__, is defined as; ${\omega }_{{v}_{j}}={v}_{i}\in {G}_{{v}_{j}}^{-}\hspace*{1em}\mid \hspace*{1em}({s}_{{v}_{i}}\geq {\tau }_{{v}_{i},{v}_{j}}\hspace*{1em}\text{and}\hspace*{1em}{\sigma }_{{v}_{i},{v}_{j}}=+)\hspace*{1em}\text{or}\hspace*{1em}({s}_{{v}_{i}}\lt {\tau }_{{v}_{i},{v}_{j}}\hspace*{1em}\text{and}\hspace*{1em}{\sigma }_{{v}_{i},{v}_{j}}=-)$.

Definition 5 **Parameters of a BRN**

The logical parameters of a BRN are indexed by its set of resources. The parameter set is a Cartesian product of each variable’s resources and its elements are of the form *K*_*ω*__*v*_*i*__. The evolution from one qualitative state to another state is determined by an evolution operator which compares discrete values of resources and parameters.

Definition 6 **Evolution Operator, Bernot et al. (2007)↱**

Let *s*_*v*_*i*__ ∈ ℕ and *K*_*ω*__*v*_*i*__ ∈ ℕ, the evolution operator (↱) is defined as follows;

(1) \begin{eqnarray*}{s}_{{\nu }_{i}}\Rsh {{K}_{\omega }}_{{v}_{i}}= \left\{ \begin{array}{@{}lll@{}} \displaystyle {s}_{{v}_{i}}+1&\displaystyle \text{iff}&\displaystyle {s}_{{v}_{i}}\lt {K}_{{\omega }_{{\nu }_{i}}}\\ \displaystyle {s}_{{v}_{i}}-1&\displaystyle \text{iff}&\displaystyle {s}_{{v}_{i}}\gt {K}_{{\omega }_{{\nu }_{i}}}\\ \displaystyle {s}_{{v}_{i}}&\displaystyle &\displaystyle \text{otherwise}. \end{array} \right. \end{eqnarray*}

Definition 7 **State Graph**

*Let *G* = (*V*, *E*) be a BRN and *s*_*v*_*x*__ is expression level of *v*_*x*_ in a state *s* ∈ *S*. Then the state graph *R* = (*S*, *T*) is a directed graph, where *S* represents set of states, and *T*⊆ *S* × *S* is a relation between states, also called the transition relation, such that *s* → *s*′ ∈ *T* iff*:

• ∃ a unique *v*_*x*_*ϵV* such that ${s}_{{v}_{x}}\not = {s}_{{v}_{x}}^{{^{\prime}}}$ and ${s}_{{v}_{x}}^{{^{\prime}}}={s}_{{v}_{x}}\Rsh {K}_{x}({\omega }_{{v}_{x}})$, and
• ∀ ${v}_{y}\epsilon V\setminus \{x\}{s}_{{v}_{y}}^{{^{\prime}}}={s}_{{v}_{y}}$.”

### Parameter inference using model checking

The dynamics of Thomas’s method are generated by translating the interaction graph to a state transition graph using a set of logical parameters, which are not known in advance. The estimation of model parameters constitutes an important step in qualitative modeling of biological networks. Bernot et al. (2004) introduced a method to decipher these parameters by employing a formal verification approach, called model checking. In this approach, known experimental observations are encoded in a temporal logic framework, called Computation Tree Logic (CTL), and then using the model checker, different parameter combinations are evaluated to finally select parameters which satisfy CTL observations. In CTL, experimental observations are encoded into formulas by using a set of quantifiers which define criteria to explore different states or paths originating from a given state. Here, we provide a brief description of these quantifiers, the detailed semantics of which can be found in Clarke, Grumberg & Peled (1999).

• **A**: This is a path quantifier which enforces that a given property should hold in all paths originating from the given state. The quantifier itself is read as “For all paths.”
• **E**: Known as the “Existential Quantifier,” this is also a path quantifier which enforces that a given property must hold in at least one path originating from the given state. The quantifier is read as: “There exists a path.”
• **G**: This quantifier is known as the “Global Quantifier” and is a state quantifier which enforces that a property holds in all states of a path originating from the given state, inclusive of the given state as well. It is read as: “Globally.”
• **F**: The “Future Quantifier” is the second state quantifier and enforces that a given property must hold in one of the future states in the path originating from the given state. It is read as: “In future” or “Eventually.” The Future Quantifier also covers the current/given state as well when checking the property.
• **X**: The “Next Quantifier” is the third state quantifier and enforces that a given property must hold in the immediate successor state. It is read as: “Next.”

SMBioNet (Khalis et al., 2009; Bernot et al., 2004) is a tool for the parameter estimation of biological networks, based on the qualitative formalism of René Thomas (Thomas, 1978; Atkinson, 1965). Given a model of a BRN in the form of Thomas’s network and behavioral properties (observations), expressed as CTL formulas, SMBioNet exhaustively enumerates all compatible parameterizations by generating a state graph for each parameter combination and by verifying the formulas on each state graph. The verification of the CTL property is performed by invoking model checker NuSMV (Cimatti et al., 2002). The parameter combinations are reduced by applying Snoussi and observability constraints (Snoussi & Thomas, 1993). Finally, all the models that satisfy the CTL properties are shortlisted. SMBioNet has been applied in studies such as: tail resorption in tadpole metamorphosis (Khalis et al., 2009), and immunity control in bacteriophage lambda (Richard, Comet & Bernot, 2006).

### Network analysis

Graph Theory (Bondy & Murty, 1976) plays an important role in the modeling and analysis of processes in several application areas, including systems biology (Pavlopoulos et al., 2011; Barabasi & Oltvai, 2004; Mason & Verwoerd, 2007). The graph-theoratic approaches are employed to analyze topological and structural parameters of biological networks to discover key properties that provide meaningful insights into the functionality of biological systems. Identification of important nodes in a large biological regulatory network is critical in the understanding of cellular mechanisms. The most widely used measure to compute the ranking of nodes in graph-theoratic models, based on the concept of Centrality (Aittokallio & Schwikowski, 2006; Mason & Verwoerd, 2007), mainly originate from Social Network Analysis (Wasserman, 1994). Centrality Analysis has also been employed to investigate important properties of complex biological regulatory networks (Koschützki & Schreiber, 2008).

Definition 8 **Betweenness Centrality**

*For a state graph *R* = (*S*, *T*) of an interaction graph *G* = (*V*, *E*), let *x*, *y* and *z* be the distinct qualitative states in $\mathcal{R}$, and let *σ*_*x*,*y*_ be the total number of trajectories from state *x* to state *y*, and let *σ*_*y*,*x*_ be the total number of trajectories from qualitative state *y* to *x*, passing through a state *z*. Let ${\mathcal{O}}_{x}$ represents the set of all ordered pairs, (*y*, *x*) such that *x*, *y* and *z* are all distinct. Then, the *Betweenness Centrality* of the qualitative state *z* can be computed from Eq. (2)*:

(2) \begin{eqnarray*}{C}_{b}(z)=\sum _{\begin{array}{@{}l@{}} \displaystyle (x,y)\in \mathcal{O} \end{array}} \frac{{\sigma }_{x,y}(z)}{{\sigma }_{x,y}} .\end{eqnarray*}

### Hybrid modeling with delays

Discrete modeling provides useful insights into qualitative dynamics of biological networks. However, an increase or decrease in protein expression, described by a step function, is not coherent with actual changes in protein expression taking place within a cell. The concentration level of a protein, for instance, does not jump from one discrete value to another discrete value. In order to capture the sigmoidal change of protein expressions, Ahmad et al. (2007) introduced a new framework based on piece-wise linear equations. In this framework, states of a system are modeled as discrete locations. Additionally, specialized variables, called clocks, are used to specify constraints for transition from one discrete location to another (Fig. 3). Here, we provide a brief description of the hybrid modeling framework, adopted from Ahmad et al. (2007) and Aslam et al. (2014).

“Clock variables are used to measure the ‘delays’ (the time duration) that needs to pass between two consecutive expression levels. Thus, a clock variable *h* is associated with each protein in the BRN. The initial values of each *h* are set to zero, which then approach either *d*^+^ or *d*^−^. *d*^+^ signifies a production delay, that is, the delay required to increase the concentration level of the associated protein by 1. Similarly, *d*^−^ signifies the degradation delay, that is, the delay to decrease the protein concentration by a single level. The rate of evolution of each *h* is given by the first order derivative *dh*∕*dt* = *r* where *r* ∈ {0, 1, − 1} (Ahmad et al., 2007).

In most cases, the exact values of the delays associated with the proteins are not known, which is why unvalued parametric delays are used. Thus, the hybrid model was constructed using the Parametric Bio Linear Hybrid Automaton (Ahmad, 2009) defined below.

Let *C*^=^(*X*, *P*), *C*^≤^(*X*, *P*), and *C*^≥^(*X*, *P*) be the set of constraints using only =, ≤, and ≥, respectively. Here, *X* and *P* are the sets of real valued variables and parameters, respectively.

Definition 9 **Parametric Bio Linear Hybrid Automaton (Bio-LHA)**

*A parametric Bio Linear Hybrid Automaton 𝔹 is a tuple (*L*, *l*_0_, *X*, *P*, *E*, Inv, Dif) where:*

• **L* is a finite set of locations*,
• **l*_0_ ∈ *L* is the initial location*,
• **P* is a finite set of parameters (delays)*,
• **X* is a finite set of real-valued variable (clocks)*,
• **E*⊆ *L* × *C*^=^(*X*, *P*) × 2^*X*^ × *L* is a finite set of edges with typical element *e* = (*l*, *g*, *R*, *l*′) ∈ *E* representing an edge from *l* to *l*′ with guard *g* and the reset set *R*⊆ *X*. The set of clocks *g* ∈ *R**,
• **Inv*:*L* → *C*^≤^(*X*, *P*)∪*C*^≥^(*X*, *P*) assigns an invariant to any location*,
• **Dif*:*L* × *X* → { − 1, 0, 1} maps each pair (*l*, *h*) to an evolution rate*.

The Transition System related semantics of the parametric Bio-LHA are given below according to the time domain 𝕋, where 𝕋^∗^ = 𝕋∖{0}.

Definition 10 **Semantics of Bio-LHA**

*Let *γ* be a valuation for the parameters *P* and *ν* represents the values of clocks in a location. The (𝕋, *γ*)-semantics of a parametric Bio-LHA 𝔹 = (*L*, ℓ_0_, *X*, *P*, *E*, Inv, Dif) is defined as a timed transition system **_*B*_ = (𝕊, *s*_0_, 𝕋, → ) where: (1) 𝕊 = {(ℓ, *ν*)|ℓ ∈ *L*and*ν*⊧Inv(ℓ)}; (2) *s*_0_ is the initial state and (3) the relation →⊆ 𝕊 × 𝕋 × 𝕊 is defined for *t* ∈ 𝕋 as*:

• *discrete transitions: (ℓ, *ν*)→^0^(ℓ′, *ν*′) if ∃(ℓ, *g*, *R*, ℓ′) ∈ *E* such that *g*(*ν*) = *true*, *ν*′(*h*) = 0 if *h* ∈ *R* and *ν*′(*h*) = *ν*(*h*) if *h*∉*R**.
• *continuous transitions: For *t* ∈ 𝕋^∗^, (ℓ, *ν*)→^*t*^(ℓ′, *ν*′) if ℓ′ = ℓ, *ν*′(*h*) = *ν*(*h*) + Dif(ℓ, *h*) × *t*, and for every *t*′ ∈ [0, *t*], (*ν*(*h*) + Dif(ℓ, *h*) × *t*′)⊧Inv(ℓ), where ⊧ represents satisfaction operator*.

Using the semantics of the Bio-LHA, Ahmad et al. (2007) then defined the temporal state space and the invariance kernel set which have been adapted below.

Definition 11 **Temporal Zone**

Temporal zone is defined as a region where time elapses until a discrete transition between states takes place.

Definition 12 **Temporal State Space**

The temporal state of a BRN is composed of the complete set of temporal zones derived from the discrete model of the said BRN.

In the hybrid model of the BRN, we denote *ϕ*(*t*) for *t* ∈ ℝ ≥ 0, while the sequence of points of a trajectory and the set of all points in the state space is denoted by *S*. A particular trajectory is said to be viable if it remains within a prescribed region known as its viability domain. The state pace is denoted by *S*. A particular trajectory is said to be viable if it remains within a prescribed region known as its viability domain.

Definition 13 **Invariance Kernel**

*A trajectory *ϕ*(*t*) is said to be viable in S if *ϕ*(*t*) ∈ *S*∀*t* ≥ 0. A subset *K* of *S* is the invariant if for any point *p* ∈ *K*, a trajectory starting in *p* is viable in *K*. An invariance kernel *K* is the largest invariant subset of *S*.* ”

## Results

### Model construction

**Figure 4:**
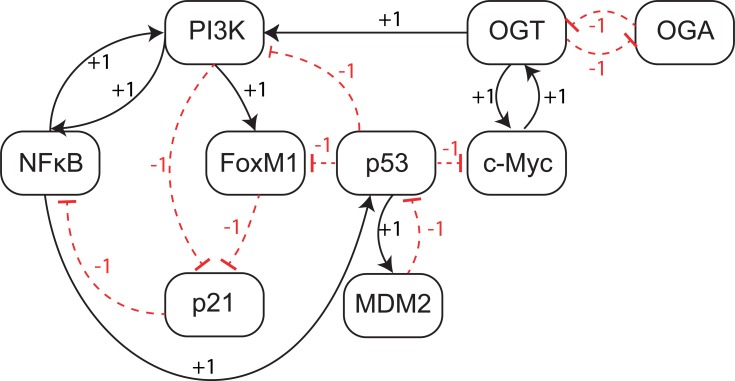
Qualitative Biological Regulatory Network (BRN). The entities are shown as nodes whereas interactions between two entities are represented with arrows. There are two types of interactions: activations (black arrows) and inhibitions (red dashed heads).

From study of the existing literature, we construct a qualitative BRN comprised of nine genes and seventeen interactions (Fig. 4). The BRN is composed of a set of well-known regulatory motifs, each of which give rise to a specific functionality of the system. First, we observe an inhibitory set of genes (OGT vs OGA) that produces a positive feedback loop, known to generate multiple stable states. In practice, a positive feedback circuit is comprised of an even number of negative elements (Plahte, Mestl & Omholt, 1995), and acts as a toggle-switch in which only one of the two genes is expressed at a time (Gardner, Cantor & Collins, 2000). On the other hand, a negative circuit is comprised of an odd number of negative interactions, such as the interaction between p53 and Mdm2, that leads to a periodic behavior or homeostasis. An important oscillatory behavior is characterized by two nested regulatory modules involving PI3K: (i) a positive feedback loop via p21 and NF-*κ*B; and (ii) a positive feedback loop between FoxM1, p21 and NF-*κ*B. The logical analysis of these regulatory motifs provides useful information about the potential behavior of a system. However, functional dynamics of a complex system, that involve both positive and negative circuits, can only be rendered with proper parameter values.

**Table 1:** Values of logical parameters estimated by using SMBioNet. The first column (Sr) indicates the serial number of each parameter with respect to its order of appearance in the SMBioNet input file (Supplemental Information 1). Each parameter is listed along with resources and permissible expression levels in second, third and fourth column, respectively. The fifth column (Selected) shows final values of logical parameters, computed by using SMBioNet.

Sr.	Parameter	Resource	Range	Selected	Sr.	Parameter	Resource	Range	Selected
1	*K*_NF*κ*B_	{}	[0]	0	21	*K*_P21_	{p53}	[0-1]	0
2	*K*_NF*κ*B_	{P21}	[0-1]	0	22	*K*_P21_	{PI3K}	[0-1]	0
3	*K*_NF*κ*B_	{PI3K}	[0-1]	0	23	*K*_P21_	{FoxM1,p53}	[0-1]	1
4	*K*_NF*κ*B_	{P21,PI3K}	[1]	1	24	*K*_P21_	{p53,PI3K}	[0-1]	1
5	*K*_PI3K_	{}	[0]	0	25	*K*_P21_	{FoxM1,PI3K}	[0-1]	1
6	*K*_PI3K_	{NF*κ*B}	[0-1]	0	26	*K*_P21_	{FoxM1,p53,PI3K}	[0-1]	1
7	*K*_PI3K_	{OGT}	[0-1]	1	27	*K*_FoxM1_	{}	[0]	0
8	*K*_PI3K_	{p53}	[0-1]	0	28	*K*_FoxM1_	{p53}	[0-1]	0
9	*K*_PI3K_	{NF*κ*B,OGT}	[0-1]	1	29	*K*_FoxM1_	{PI3K}	[0-1]	1
10	*K*_PI3K_	{OGT,p53}	[0-1]	1	30	*K*_FoxM1_	{p53,PI3K}	[1]	1
11	*K*_PI3K_	{NF*κ*B,p53}	[0-1]	1	31	*K*_OGT_	{}	[0]	0
12	*K*_PI3K_	{NF*κ*B,OGT,p53}	[1]	1	32	*K*_OGT_	{C-Myc}	[0-1]	1
13	*K*_OGA_	{}	[0]	0	33	*K*_OGT_	{OGA}	[0-1]	0
14	*K*_OGA_	{OGT}	[1]	1	34	*K*_OGT_	{C-Myc,OGA}	[1]	1
15	*K*_p53_	{}	[0]	0	35	*K*_Mdm2_	{}	[0]	0
16	*K*_p53_	{Mdm2}	[0-1]	1	36	*K*_Mdm2_	{p53}	[1]	1
17	*K*_p53_	{NF*κ*B}	[0-1]	1	37	*K*_C-Myc_	{}	[0]	0
18	*K*_p53_	{Mdm2,NF*κ*B}	[0-1]	1	38	*K*_C-Myc_	{OGT}	[0-1]	1
19	*K*_P21_	{}	[0]	0	39	*K*_C-Myc_	{p53}	[0-1]	0
20	*K*_P21_	{FoxM1}	[0-1]	0	40	*K*_C-Myc_	{OGT,p53}	[1]	1

### Logical parameters

Table 1 enlists the final values of logical parameters which are used to generate model trajectories as a state transition graph. These parameters are computed from known qualitative observations expressed as CTL formulas (Eqs. (3)–(6)), the most important of which is the change in expression levels of OGT and OGA in different types of cancers, including tumors of the breast and colon (Singh et al., 2015; Fardini et al., 2013; De Queiroz, Carvalho & Dias, 2014). The sub-formulas *ψ*_1_ and *ψ*_2_ represent changes in the HBP pathway, triggered by a change in the expression level of two genes: OGT and OGA. The sub-formula *ψ*_1_ describes behavior of the biological system under enhanced OGT expression leading to a future state in which the expression of oncogenes remain high. The sub-formula *ψ*_2_ describes that there is at-least one trajectory in which expression of oncogenes remain low when OGT is initially not expressed. Finally, the sub-formula *ψ*_3_ represents oscillatory behavior exhibited by the HBP pathway and tumor suppressor proteins p53 and p21. We used SMBioNet software (Bernot et al., 2004; Khalis et al., 2009) to select only those parameters that satisfy the CTL formulas. SMBioNet selected four models (Supplemental Information 2 and 3) which show a single deadlock state (1,0,1,1,1,1,1,0,1) and plausible biological trajectories in cancer progression and recovery.

(3) \begin{eqnarray*}\begin{array}{@{}l@{}} \displaystyle {\psi }_{1}=((\mathrm{OGT}=1\wedge \mathrm{OGA}=0)\Rightarrow \mathbf{EF}(\mathbf{AG}(\mathrm{OGT}=1\wedge \\ \displaystyle \hspace*{15.0pt}\mathrm{OGA}=0\wedge \mathrm{PI3K}=1\wedge \mathrm{FOXM}=1\wedge \mathrm{P21}=0\wedge \mathrm{CMyc}=1))) \end{array}\end{eqnarray*}

(4) \begin{eqnarray*}\begin{array}{@{}l@{}} \displaystyle {\psi }_{2}=((\mathrm{OGT}=0\wedge \mathrm{OGA}=1)\Rightarrow \mathbf{EF}(\mathbf{AG}(\mathrm{OGT}=0\wedge \\ \displaystyle \hspace*{15.0pt}\mathrm{OGA}=1\wedge \mathrm{PI3K}=0\wedge \mathrm{FOXM}=0\wedge \mathrm{P21}=1\wedge \mathrm{CMyc}=0))) \end{array}\end{eqnarray*}

(5) \begin{eqnarray*}\begin{array}{@{}l@{}} \displaystyle {\psi }_{3}=((\mathrm{OGT}=0\wedge \mathrm{OGA}=1\wedge \mathrm{p53}=1\wedge \mathrm{P21}=1)\Rightarrow \\ \displaystyle \hspace*{15.0pt}\mathbf{EX}(\mathbf{EF}(\mathrm{OGT}=0\wedge \mathrm{OGA}=1\wedge \mathrm{p53}=1\wedge \mathrm{P21}=1))) \end{array}\end{eqnarray*}

(6) \begin{eqnarray*}\begin{array}{@{}l@{}} \displaystyle \mrm{\Phi }=({\psi }_{1})\wedge ({\psi }_{2})\wedge ({\psi }_{3}). \end{array}\end{eqnarray*}

The tendency of each gene to change its expression level is a function of presence or absence of its resources. The change in expression level of a gene can be determined by comparing its current state, at any particular time, with values of logical parameters listed in Table 1. The inferred parameters indicate that in the presence of the PI3K activation signal, NF*κ*B maintains a higher expression level (if already expressed i.e., 1) or shows an increase in its expression level. On the other hand, PI3K shows a rise in expression even in the presence of a p53 inhibition signal when both NF*κ*B and OGT are activating PI3K. The expression level of tumor suppressor protein p53 shows an increase only in the absence of the MDM2 inhibition signal. The inferred parameters show increase in expression level of OGT and C-Myc when there is an activation signal between them. The collective behavior of genes involved in a biological system can only be determined only by analyzing trajectories in a state transition graph.

**Figure 5:**
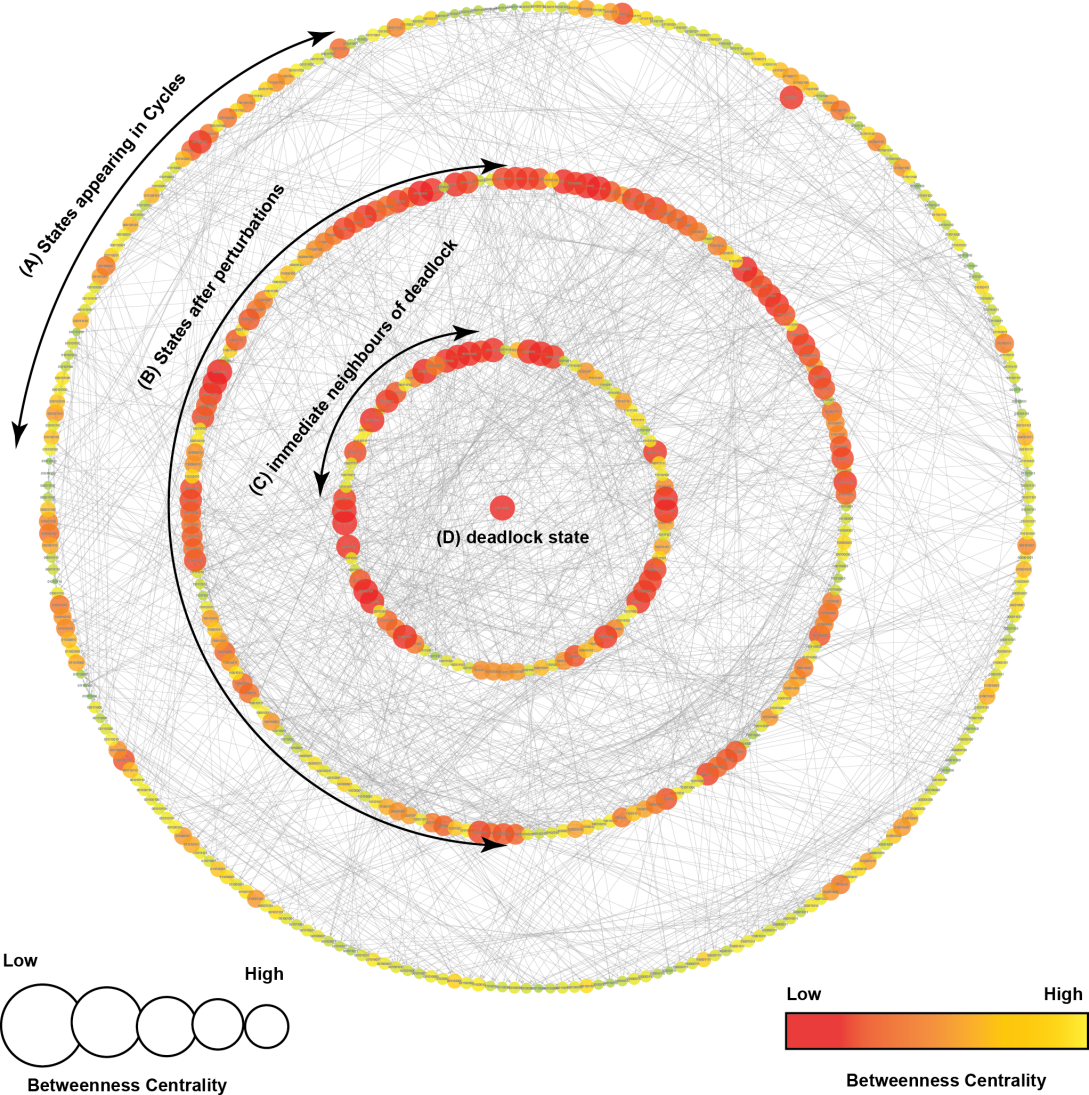
State Graph (rendered using Cytoscape) with 512 nodes and 2,304 edges. Each node in the graph, shown as a circle, represents a unique state characterized by the expression levels of individual genes. The size and color of each state is defined based on its betweenness centrality. (A) This outermost circle represents the states appearing in cycles. (B) Once the system is perturbed, it diverges to several bifurcation states that can either lead to deadlock or recovery, dependent on signaling events. Compared to the outermost circle, (C) neighboring states and (D) the deadlock state have lower betweenness centrality.

### State transition graph

A state transition graph of the biological regulatory network with 512 nodes and 2,304 edges (Supplemental Information 4) is rendered using Cytoscape software (Shannon et al., 2003) (Fig. 5). The graph is generated from selected logical parameters (Table 1) using GENOTECH software (Ahmad, 2009; Aslam et al., 2014; Ahmad et al., 2012) and states are sorted on the basis of betweenness centrality. A parameterized BRN is also attached for the GINsim Tool software (Supplemental Information 8) (Chaouiya, Naldi & Thieffry, 2012). The deadlock state (1,0,1,1,1,1,1,0,1) (Fig. 5D) shows high expression levels of OGT and oncogenes. The immediate predecessors (up to two levels) of the deadlock state have low betweenness centrality, indicated with circles, having comparatively larger diameters and darker colors. The model also shows several cycles, and rendered as an outermost circle (Fig. 5). These states have high betweenness centrality, represented using circles with smaller diameters and lighter colors. The cycles demonstrate normal behaviors of the system characterized by low expression levels of oncogenes, oscillation of the p53-Mdm2 circuit, and moderate expression levels of tumor suppressor proteins. In the state graph, the state of the system at a particular time is represented by a vector containing expression levels of all entities. The normal state is characterized by low expression levels of OGT, PI3K, and FoxM1 along with the presence of tumor suppressor proteins p53 and p21. This state is represented as a vector (OGT = 0, OGA = 1, PI3K = 0, FoxM1 = 0, p53 = 1, p21 = 1). Conversely, the pathogenic state is characterized by high expression levels of OGT along with PI3K and FoxM1. (OGT = 0, OGA = 1, PI3K = 0, FoxM1 = 0, p53 = 1, p21 = 1) represents a normal or recovery state of the system. Biological systems, under normal circumstances, exhibit oscillatory behavior or homeostasis during which the overall state of the system remains in a cycle of normal states. Therefore, the desirable qualitative model should exhibit pathogenic trajectories along with normal homeostatic behavior represented as a cycle or closed path. The normal behavior, characterized by low expression levels of OGT and the presence of OGA, is encoded as CTL formula *ψ*_2_. It states that, under normal circumstances, when the expression level of OGT is low, the system will always remain in a stable state characterized by low expression of oncogenes (OGT, FoxM1, PI3K, c-Myc) and the presence of tumor suppressors (p53 and p21).

**Figure 6:**
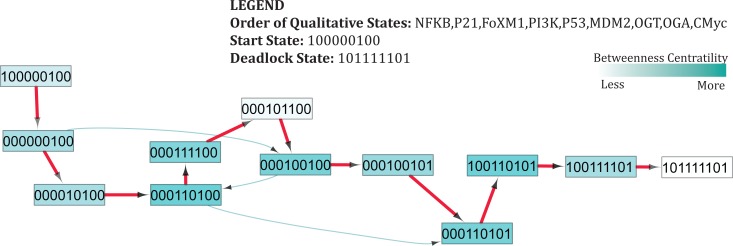
Subgraph isolated from the state transition graph (Fig. 5), highlighting tumor progression from the start state (1,0,0,0,0,0,1,0,0) leading to the deadlock state (1,0,1,1,1,1,1,0,1). Each node in the graph represents a unique state of the system characterized by qualitative expression of genes in the following order: NF-*κ*B, p21, FoxM1, PI3K, p53, MDM2, OGT, OGA, c-Myc. Activation of a particular gene/entity is indicated with “1”, whereas “0” indicates that the expression level of a gene is below the activation threshold. Nodes are shaded based on the level of betweenness centrality. Nodes and trajectories associated with tumor progression and recovery are denoted using red and green arrows, respectively.

**Figure 7:**
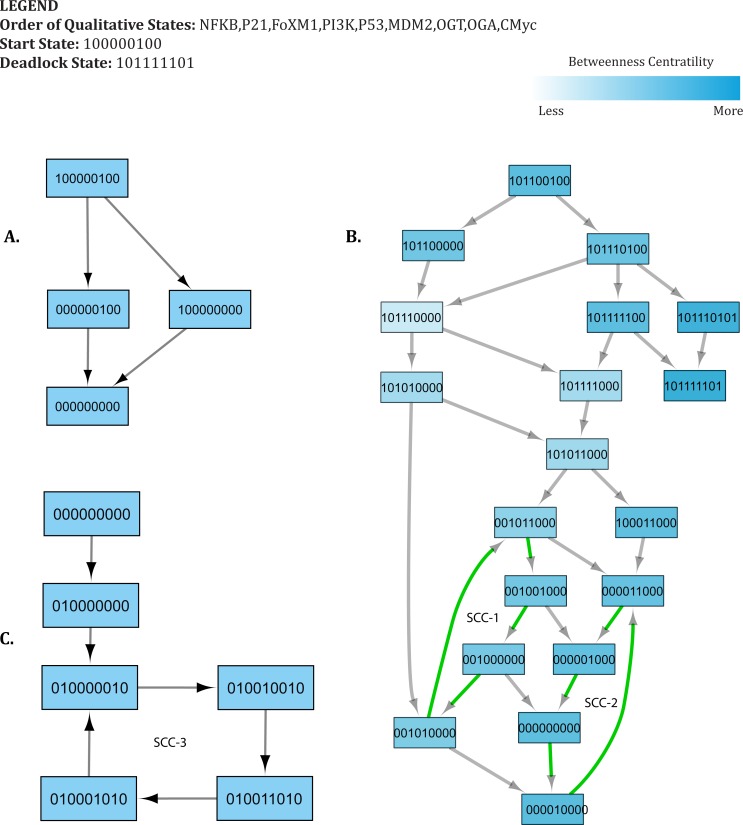
Important trajectories involved in Recovery. Each node in the graph represents a unique state of the system characterized by the expression level of genes in the following order: NF-*κ*B, p21, FoxM1, PI3K, p53, MDM2, OGT, OGA, c-Myc. Activation of a particular gene/entity is indicated with “1”, whereas “0” indicates that the expression level of a gene is below the activation threshold. Nodes are shaded based on the level of betweenness centrality. Nodes and trajectories involved in oscillations are denoted using green arrows. SCC highlights strongly connected components. (A) Transition from the start state (1,0,0,0,0,0,1,0,0) to the recovery state (0,0,0,0,0,0,0,0,0). (B) Possible transition from advanced tumor progression to the recovery state. (C) Transition from the recovery state (0,0,0,0,0,0,0,0,0) to attractor. The deadlock state is represented as (1,0,1,1,1,1,1,0,1).

The graphs presented in Figs. 6–7 are sub-graphs, extracted from the complete state graph and show step-by-step changes towards progression of cancer and recovery, respectively. Since the complete state graph obtained from qualitative modeling is too complex to analyze each trajectory manually, we used the idea of betweenness centrality to identify important trajectories.

### HyTech results

**Figure 8:**
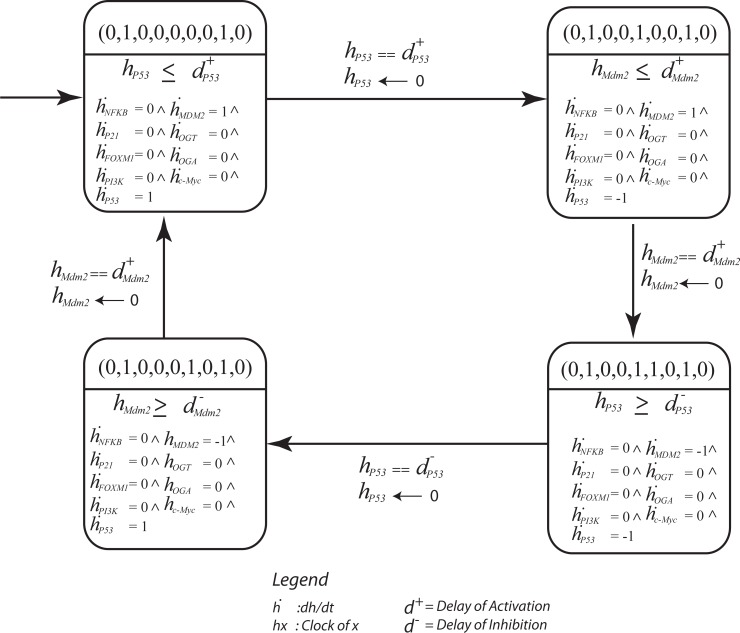
The parametric Bio Linear Hybrid Automaton (Bio-LHA) of the significant cycle (0, 1, 0, 0, 0, 0, 0, 1, 0) → (0, 1, 0, 0, 1, 0, 0, 1, 0) → (0, 1, 0, 0, 1, 1, 0, 1, 0) → (0, 1, 0, 0, 0, 1, 0, 1, 0) → (0, 1, 0, 0, 0, 0, 0, 1, 0). The states are represented by a vector containing expression level of genes in the order: Nf*κ*B, P21, FoXM1, Mdm2, OGT, OGA, C-Myc. The significant cycle is characterized by low expression of OGT and oncogenes, and oscillatory behavior of the p53-Mdm2 circuit resulting in homeostasis. Here, each location represents a qualitative state of the cycle with invariants (conjunction of constraints) and clock rates. The transitions are labeled with guards and the clock resets of evolving entities. The initial state (0,0,0,0,0,0,0,0,0) is represented by a small arrow shown in upper left corner.

Hybrid modeling was carried out using the HyTech (HYbrid TECHnology) tool (Henzinger, Ho & Wong-Toi, 1997). The Bio-LHA of the significant cycle [(0, 1, 0, 0, 0, 0, 0, 1, 0) → (0, 1, 0, 0, 1, 0, 0, 1, 0) → (0, 1, 0, 0, 1, 1, 0, 1, 0) → (0, 1, 0, 0, 0, 1, 0, 1, 0) → (0, 1, 0, 0, 0, 0, 0, 1, 0)] defines invariants and clock rates for each qualitative state in the cycle (Fig. 8). The invariance kernel for the cycle (HyTech code in Supplemental Information 6) is composed of three conjuncted delay constraints, (Table 2). These delay constraints define necessary and sufficient conditions in a way such that the resulting trajectories maintain cyclic stability (homeostasis). If these delay constraints are violated, the trajectories will deviate from the significant cycle and may follow a path to the deadlock state. In Table 3, a pair-wise matrix represents an association between these delay constraints with the help of logical relations (≤, <, >, ≥, =).

**Table 2:** Invariance Kernel of the significant cycle. The invariance kernel dictates the delay constraints that are being followed in this cycle.

Qualitative cycle	(0, 1, 0, 0, 0, 0, 0, 1, 0) → (0, 1, 0, 0, 1, 0, 0, 1, 0) → (0, 1, 0, 0, 1, 1, 0, 1, 0) →(0, 1, 0, 0, 0, 1, 0, 1, 0) → (0, 1, 0, 0, 0, 0, 0, 1, 0)	
Invariance kernel	Conjunction of constraint I–IV:	
	I.	${d}_{\mathrm{p53}}^{+}+ \left\vert {d}_{\mathrm{Mdm2}}^{-} \right\vert \leq {d}_{\mathrm{Mdm2}}^{+}+ \left\vert {d}_{\mathrm{p53}}^{-} \right\vert $
	II.	${d}_{\mathrm{Mdm2}}^{+}+ \left\vert {d}_{\mathrm{p53}}^{-} \right\vert \leq 0$
	III.	$ \left\vert {d}_{\mathrm{Mdm2}}^{-} \right\vert \leq {d}_{\mathrm{Mdm2}}^{+}+ \left\vert {d}_{\mathrm{p53}}^{-} \right\vert $

**Table 3:** Relation matrix of the significant cycle which depicts binary relations between the states. Each entry of the matrix represents whether a delay ‘a’ would be greater than, equal to, or less than a delay ‘b.’ The matrix is read as *d*_row_ ∼ *d*_column_ where ∼ ∈ { ≤ , < , > , ≥ , = }.

Relation matrix				
	$ \left\vert {d}_{\mathrm{Mdm2}}^{-} \right\vert $	$ \left\vert {d}_{\mathrm{p53}}^{-} \right\vert $	${d}_{\mathrm{Mdm2}}^{+}$	${d}_{\mathrm{p53}}^{+}$
$ \left\vert {d}_{\mathrm{Mdm2}}^{-} \right\vert $	=	= ≥ , ≤	= ≥ , ≤	= ≥
$ \left\vert {d}_{\mathrm{p53}}^{-} \right\vert $	–	=	≥	≥, ≤
${d}_{\mathrm{Mdm2}}^{+}$	–	–	=	>, <
${d}_{\mathrm{p53}}^{+}$	–	–	–	=

The delay constraints from the start state (1,0,0,0,0,0,1,0,0) to the deadlock state (1,0,1,1,1,1,1,0,1) are computed (HyTech code in Supplemental Information 7) and presented in the form of a relation matrix in Table 4, which highlight important relations between the production and degradation rates of important proteins. The degradation delay of OGT is greater than the activation delays of MDM2, PI3K and p53, meaning that during progression towards deadlock state, depletion of OGT takes place at a much slower rate compared to MDM2, PI3K and p53 (Table 4). Similarly, production delay of c-Myc remains higher than PI3K and p53-MDM2 circuitry, thus reinforcing the results of our qualitative modeling which suggest that once c-Myc and OGT form a positive feedback loop, the overall system eventually moves to the deadlock state.

**Table 4:** Relation matrix computed from delay constraints that lead to the deadlock state (1,0,1,1,1,1,1,0,1) from starting state (1,0,0,0,0,0,0,1,0,0). Each entry of the matrix represents whether a delay ‘a’ would be greater than, equal to, or less than a delay ‘b.’ The matrix is read as *d*_row_ ∼ *d*_column_ where ∼ ∈ { ≤ , < , > , ≥ , = }.

	dnOGT	dpCMYC	dpFOXM	dpP21	dpNF*κ*B	dnMDM2	dnP53	dpMDM2	dnNF*κ*B	dpPI3K	dpP53
dnOGT	=	>=	=<>	=><	>=<	>	>	>	=<>	>	>
dpCMYC		=	<=	<=	=<	>	>	>	<=>	>	>
dpFOXM			=	=><	>=<	>	>	>	=><	>	>
dpP21				=	>=<	>	>	>	=<>	>	>
dpNF*κ*B					=	>	>	>	<=>	>	>
dnMDM2						=	>=	>=	<=>	>=	>=
dnP53							=	=	<=	=	=
dpMDM2								=	<=	=	=
dnNF*κ*B									=	>=	>=
dpPI3K										=	=
dpP53											=

## Discussion

Previous studies have demonstrated a link between increased cellular O-GlcNAcylation (hyper-O-GlcNAcylation) and cancer progression in multiple tumor types (Caldwell et al., 2010; Fardini et al., 2013; Slawson, Copeland & Hart, 2010). For example, recent findings in breast cancer have shown that breast tumor tissues and cell lines have increased mRNA expression of OGT and decreased mRNA expression of mRNA as compared to the adjacent normal (Krześlak et al., 2012a; Caldwell et al., 2010). Additional studies have observed that reduction of OGT expression in prostate cancer cells inhibits metastatic tumor progression to bone (Lynch et al., 2012). Similarly in lung and colon tumor tissues, OGT expression was elevated compared with surrounding normal tissue (Mi et al., 2011). Importantly, our present quantitative model validates the critical role of OGT in regulating cancer development and recovery in multiple tumor types.

### Qualitative dynamics

The qualitative model, rendered as a state transition graph (Fig. 5), highlights important behaviors in the form of trajectories to represent temporal evolution of the overall system from one qualitative state to another. These behaviors mainly include cycles that represent homeostasis, stable states (deadlock), and several bifurcation states from where the system can evolve either in the direction of tumor invasion or recovery. Three bifurcation states [(1,0,1,1,0,0,1,0,0), (1,0,1,1,1,0,1,0,0), and (1,0,1,1,1,1,1,0,0)] were observed to lead to both a deadlock state (1,0,1,1,1,1,1,0,1) and a typical reset state (0,0,0,0,0,0,0,0,0). Network analysis carried out using Cytoscape (Shannon et al., 2003) helped to identify important trajectories based on betweenness centrality and highlight step-by-step alterations that systematically lead to cancer metastasis from the starting state. Here, we discuss two cases that focus on important trajectories of the qualitative model involved in tumor progression and recovery.

### Case 1: cancer initiation and progression

Under normal physiological conditions, proto-oncogenes play basic roles in signaling pathways that control cellular growth (Pall, 1981). Activation of a proto-oncogene into an oncogene, through gain-of-function mutations, increases the expression of these proteins and leads to alterations in signaling pathways, increases glycolytic flux through the HBP, and elicits an inflammatory response (Ma, Vocadlo & Vosseller, 2013; Karin, 2009; Fardini et al., 2013). Important trajectories that originate from a starting state (1,0,0,0,0,0,1,0,0) show increased expression of the pro-inflammatory NF-*κ*B pathway and O-GlcNAc transferase (OGT) (Fig. 6). Supplemental Information 9 highlights changes in the expression level of genes along each transition that lead to the deadlock state. Enhanced OGT expression is considered an indicator of metabolic switch from oxidative phosphorylation to glycolysis, and can occur under oxidative stress. Cellular response to this stress has been shown to activate a p53 transcriptional response (Gambino et al., 2013), leading to a qualitative state (1,0,0,0,1,0,1,0,0). The subsequent trajectories, mapped in Fig. 6, show that p53 is over-expressed in several states that lead to a deadlock state, thereby reinforcing the growing evidence that suggests the divergent role of p53 in response to increased cellular metabolism (Puzio-Kuter, 2011; Maddocks & Vousden, 2011).

Despite its original classification as a tumor suppressor gene, recent evidence is accruing to reveal p53 also carries oncogenic properties (Soussi & Wiman, 2015). The majority of p53 germline and somatic alterations are missense mutations which synthesizes a stable mutant p53 protein that accumulates in the nucleus of tumor cells and can result in an oncogenic phenotype (Dittmer et al., 1993). Indeed, over-expression of p53 has been reported in various breast cancer studies, and induced a metabolic shift toward glycolysis (Won et al., 2012). Similar findings have also been reported in other tumor types, including cancers of the colon, cervix, and pancreas (Al-Khayal et al., 2016; Rajeshkumar et al., 2015; Hernández-Reséndiz et al., 2015; Kruiswijk, Labuschagne & Vousden, 2015).

In addition to accumulation of p53, significant increase in the expression of NF-*κ*B has been reported in different tumor types, including breast and prostate cancers (Arora et al., 2014; Mak et al., 2015). Recent studies have shown the anti-apoptotic properties of hyper O-GlcNAcylation in tumor cells and the contribution of this post-translational modification for oncogenic activation of NF-*κ*B in pancreatic cancers (Ma & Vosseller, 2013). In corroboration with these studies, our qualitative model demonstrates a sustained activation of NF-*κ*B, which contributes to increased glycolytic flux and tumorigenesis.

The qualitative model reveals activation of FoxM1 and PI3K in response to continuous activation of OGT, thus leading to qualitative states (1,0,0,1,0,1,0,0) and (1,1,1,1,1,0,1,0,0). As expected, FoxM1 over-expression has been implicated in cancer. Although the mechanism of FoxM1 and OGlcNAcylation is poorly characterized, sentinel studies suggest that hyper O-GlcNAcylation of FoxM1 mediators in breast cancer prevent the degradation of FoxM1, to promote transformation of cells in breast cancer (Caldwell et al., 2010). From the initial state, betweenness centrality of states decreases as systems get closer to the deadlock state (Fig. 6). This drop in betweenness centrality is indicative of fewer chances for transition to recovery, particularly once FoxM1 and PI3K are over-expressed. Importantly, these results highlight the critical role of c-Myc to reach the deadlock state in the qualitative model. Prior to the activation of c-Myc, several bifurcation states exist for possible transitions to a typical reset or recovery state (0,0,0,0,0,0,0,0,0). However, qualitative modeling demonstrates that activation of c-Myc promotes the stability of hyper- O-GlcNAcylation. We observed that c-Myc activation forms a positive feedback loop with OGT, which plays a critical role in uncontrolled proliferation of tumor cells. Our findings are consistent with current literature that hypoxia-inducible factor (HIF), a transcription factor that activates aerobic glycolysis under cellular stress, cooperates with c-Myc to flip the metabolic switch and fuel glycolysis (Semenza, 2007; Dang et al., 2008; Kroemer & Pouyssegur, 2008). Together these proteins also upregulate glucose transporters, glycolytic intermediates, and induce angiogenesis in the tumor microenvironment to maintain glycolytic conditions (Kroemer & Pouyssegur, 2008).

Constitutive activation of c-Myc and p53 mutation contribute to uncontrolled cellular proliferation associated with upregulated glycolysis and metabolic re-programming in tumors. Further, activation of NF-*κ*B downregulates oxidative phosphorylation in various tumor types (Markert, Levine & Vazquez, 2012). Together, our findings further characterize the critical roles of these oncogenes and tumor suppressor genes in support cancer progression through the regulation of biological networks.

### Case 2: recovery from advanced tumor progression

In our qualitative model, important trajectories involved in cancer recovery suggest that the p53-Mdm2 circuit undergoes a series of cycles first to restore the system back to recovery state and subsequently, maintain homeostasis. These cycles are depicted as strongly connected components (SCC) in Fig. 7 (SCC1, SCC2, and SCC3). Supplemental Information 10 highlights change in expression level of genes along each transition that lead to the recovery state. In Fig. 7, the initial state (1,0,1,1,0,0,1,0,0) is a bifurcation state characterized by high expression of oncogenes and increased glycolytic flux. The initial state may lead to both recovery and deadlock states depending on genetic alterations regulating the expression of genes involved in signaling. The qualitative model shows that, in response to oncogene activation and increased glycolytic flux, p53 remains constitutively active in several successor states. Increased expression of p53 mediates the down-regulation of OGT by inhibiting c-Myc, thus leading to a state (1, 0, 1, 1, 0, 0, 0, 0, 0). Subsequently, down-regulation of PI3K is also triggered by p53- mediated inhibition through p21 and low expression of OGT, resulting in a qualitative state (1, 0, 1, 0, 1, 1, 0, 0, 0). In subsequent trajectories, the p53-Mdm2 circuit acts as a repair mechanism to systematically reduce the expression of oncogenes through a series of oscillations.

• The first cycle (SCC-1) shows an oscillation of the p53-Mdm2 circuit, while maintaining increased expression of FoxM1 throughout the cycle.
• In the second cycle (SCC-2), the p53-Mdm2 circuit oscillates to down-regulate the expression of FoxM1 until the system reaches to recovery state (0,0,0,0,0,0,0,0,0).

These findings are in agreement with previous experimental studies, which suggest that p53 levels showed a series of pulses in response to DNA damage. Uri Alon, in 2000, first reported that the p53-Mdm2 circuit show dampened oscillations in irradiated breast cancer cells (Bar-Or et al., 2000). Later studies confirmed these results by showing that the p53-Mdm2 circuit undergoes a series of pulses at regular intervals (Lahav et al., 2004; Lahav, 2009). The results presented in our study illustrate the role of the p53-Mdm2 circuit in a series of oscillations that lead to recovery state, consistent with previous studies (Poltz & Naumann, 2012; Abou-Jaoudé, Ouattara & Kaufman, 2009). A similar behavior is exhibited by our qualitative model in the form of two important cycles, shown as SCC-1 and SCC-2 (Fig. 7) before reaching a recovery state. However, this model does not provide any information about the number of iterations or time spent within each cycle. Depending on the extent of DNA damage, two scenarios are possible: (1) the recovery state (0,0,0,0,0,0,0,0,0) may also serve as an unperturbed stable state where the p53 level remains low; or (2) the system reaches a ‘limit cycle’/attractor (SCC-3) where it continues to oscillate indefinitely with constant time period and amplitude. In practice, the systems comprised of negative feedback loops, like circadian rhythms, are fully capable of producing sustained oscillations to maintain homeostasis.

### Hybrid modeling

Cellular metabolism and intracellular signaling converge into a complex regulatory network that is regulated by key interactions. Importantly, these interactions that regulate these pathways vary in speed. While changes in gene expression occur at a slower rate, post-translational protein modifications tend to occur rapidly (Chubukov et al., 2014). In our study, the behavior of the p53-Mdm2 circuit is dependent on the time delay between p53-dependent induction and Mdm2-controlled repression. The delay constraints computed using HyTECH for the significant cycle [(0, 1, 0, 0, 0, 0, 0, 1, 0) → (0, 1, 0, 0, 1, 0, 0, 1, 0) → (0, 1, 0, 0, 1, 1, 0, 1, 0) → (0, 1, 0, 0, 0, 1, 0, 1, 0) → (0, 1, 0, 0, 0, 0, 0, 1, 0)] with lowest betweenness centrality, serves as an important attractor. It represents normal homeostasis characterized by low expression of oncogenes and p53-Mdm2 oscillations. Therefore, it is important to know the necessary and sufficient conditions that limit the system to maintain a homeostatic behavior.

The first state observed in the cycle (0,0,0,0,0,0,0,0,0) represents an initial configuration of the system. This state also represents a typical reset state after recovery. Different trajectories have been outlined that lead to this state (Fig. 7). The results of qualitative modeling (Fig. 7) show that once the system reaches the reset state, it enters into an attractor (SCC3). This cycle [(0, 1, 0, 0, 0, 0, 0, 1, 0) → (0, 1, 0, 0, 1, 0, 0, 1, 0) → (0, 1, 0, 0, 1, 1, 0, 1, 0) → (0, 1, 0, 0, 0, 1, 0, 1, 0) → (0, 1, 0, 0, 0, 0, 0, 1, 0)], characterized by p53-Mdm2 oscillatory behavior and low expression levels of OGT and oncogenic proteins, represents the normal homeostatic behavior of the overall system. Moreover, network analysis reveals that this cycle has the lowest betweenness centrality among all the cycles in the qualitative model, which makes it an attractive cycle. Once the system enters into this cycle (attractor), it tends to limit itself only within the cycle. Therefore, it is important to compute delay constraints that enforce the system to maintain this cyclic behavior.

Table 2 presents the invariance kernel of this important cycle. It is composed of four conjuncted delay constraints, which remain true within the cycle. Apparently, (${d}_{\mathrm{p53}}^{-}\geq {d}_{Mdm2}^{+}$) is the most significant constraint which states that the degradation delay of p53 is greater than the production delay of Mdm2. That is, the rate of p53 synthesis must be greater than or equal to the production rate of Mdm2 for the system to maintain homeostasis. Experimental studies corroborate that a long time delay between the increase in p53 and the increase in Mdm2 would lead to oscillatory behavior (Tyson, 2004; Ciliberto, Novák & Tyson, 2005). Additional work demonstrated that the amplitude of oscillations in the p53-Mdm2 negative feedback loop are more variable than the period as a result of low-frequency noise in rates of protein production (Geva-Zatorsky et al., 2006). Fluctuations in the behavior of protein circuits produces biological response variations even between individual cells. In our study, we elucidated differences in the rate of cellular functions and identified the requirements needed for a system to maintain homeostasis. Taken together, these results provide a deeper understanding of the modulation of biological networks that play critical roles in tumorigenesis.

## Conclusion

Hyper O-GlcNAcylation is known to upregulate key oncogenes and play an important role in cancer metabolism and tumorigenesis. However, the precise mechanism of oncogenic activation by O-GlcNAcylation resulting in enhanced cancer progression, has not yet been clearly established. In this paper, we used a computational modeling approach to study the function of the Hexosamine Biosynthetic Pathway, which triggers hyper O-GlcNAcylation. Within the p53-Mdm2 circuit, we found that p53 synthesis must occur at a greater than or equal rate to Mdm2 production in order to restore the system to a cancer recovery state and preserve homeostasis. We analyzed different simulation trajectories, which showed that enhanced expression of O-GlcNAc-transferase (OGT) consistently upregulates NF-*κ*B, PI3K and FoxM1. Moreover, when OGT forms a positive feedback loop with c-Myc, the overall system converges to a deadlock state from where recovery is not possible. These findings suggest that OGT is acting as a critical mediator of various oncogenic and tumor suppressor proteins implicated in tumor growth and development. We acknowledge that our findings are derived from a qualitative approach and could be dependent on cellular dynamics and environment. However, these discoveries form the foundation and direction of future translational research studies to design a quantitative model with additional tools and experimental verification for the development of molecular therapeutics. Taken together, mechanism-based therapies that are designed to target hyper O-GlcNAcylation and OGT may hold clinical benefits in the treatment of cancer.

##  Supplemental Information

10.7717/peerj.2348/supp-1 Supplemental Information 1 Raw Data: Qualitative Model with Logical Parameters (SMBioNet Code)

10.7717/peerj.2348/supp-2 Supplemental Information 2 SMBioNet Output Raw Data: SMBioNet selects 4 models after scanning different combinations of logical parameters. The output of SMBioNet containins each valuation of parameter which is contained in this file.

10.7717/peerj.2348/supp-3 Supplemental Information 3 Logical Parameters Profile Comparison of parameter values and set of selected parameters are highlighted.

10.7717/peerj.2348/supp-4 Supplemental Information 4 State Graph generated from selected parameters (in SIF format)

10.7717/peerj.2348/supp-5 Supplemental Information 5 Java Code to select a path based on average betweenness centrality

10.7717/peerj.2348/supp-6 Supplemental Information 6 Hybrid Model of attractor (HyTECH Code)

10.7717/peerj.2348/supp-7 Supplemental Information 7 Hybrid Model of path involved in progression (HyTECH Code)

10.7717/peerj.2348/supp-8 Supplemental Information 8 Parameterized BRN file for GINsim Tool

10.7717/peerj.2348/supp-9 Supplemental Information 9 Subgraph isolated from the state transition graph (Fig. 5) highlights tumor progression Each node in the graph represents a unique state of the system characterized by qualitative expression of genes in the following order: (NF-*κ*B, p21, FoxM1, PI3K, p53, Mdm2, OGT, OGA, c-Myc). Activation of a particular gene/entity is indicated as “1”, whereas “0” indicates and expression level for the particular gene that is below the activation threshold. Nodes are shaded based on the level of betweenness centrality. Each transition is labelled with change in expression level of gene (”+” shows increase and ”-” shows decrease). Nodes and transitions typically associated with tumor progression are represented with arrows colored in red. The nodes and trajectories involved in recovery are represented with arrows colored in green. The trajectories start at state “100000100” and finally lead to a deadlock state (“101111101”).

10.7717/peerj.2348/supp-10 Supplemental Information 10 Important Trajectories involved in Recovery Trajectories from start state (1000000100) to recovery state (shown in A). Trajectories from advanced tumor progression to recovery state (000000000) (B). Transition from recovery state to attractor (C).The subgraph of the state transition graph highlights possible recovery from advanced tumor progression. Each node in the graph represents a unique state of the system characterized by the expression level of critical genes (NF-*κ*B, p21, FoxM1, PI3K, p53, Mdm2, OGT, OGA, c-Myc). Activation of a particular gene/entity is indicated as “1”, whereas “0” indicates and expression level for the particular gene that is below the activation threshold. Nodes are shaded based on the level of betweenness centrality. Each transition is labelled with change in expression level of gene (”+” shows increase, ”-” shows decrease). The trajectories involved in oscillations are represented with arrows colored in green. The trajectories start at state “101100100” and finally lead to either recovery (“000000000”) or a deadlock state (“101111101”).

10.7717/peerj.2348/supp-11 Supplemental Information 11 Table of the tools used in the study

## Additional Information and Declarations

### Data Availability

The following information was supplied regarding data availability: The raw data has been supplied as a Supplementary File.
