# Supplementary figures and images for: Formal modeling and analysis of the hexosamine biosynthetic pathway: role of O-linked N-acetylglucosamine transferase in oncogenesis and cancer progression
Source: PeerJ. 2016 Sep 27;4:e2348. doi: 10.7717/peerj.2348 (PMC5047222; doi:10.7717/peerj.2348)

## LEGEND

**Order of Qualitative States:** NFkB,P21,FoXM1,PI3K,P53,MDM2,OGT,OGA,CMyc

**Start State:** 100000100

**Deadlock State:** 101111101

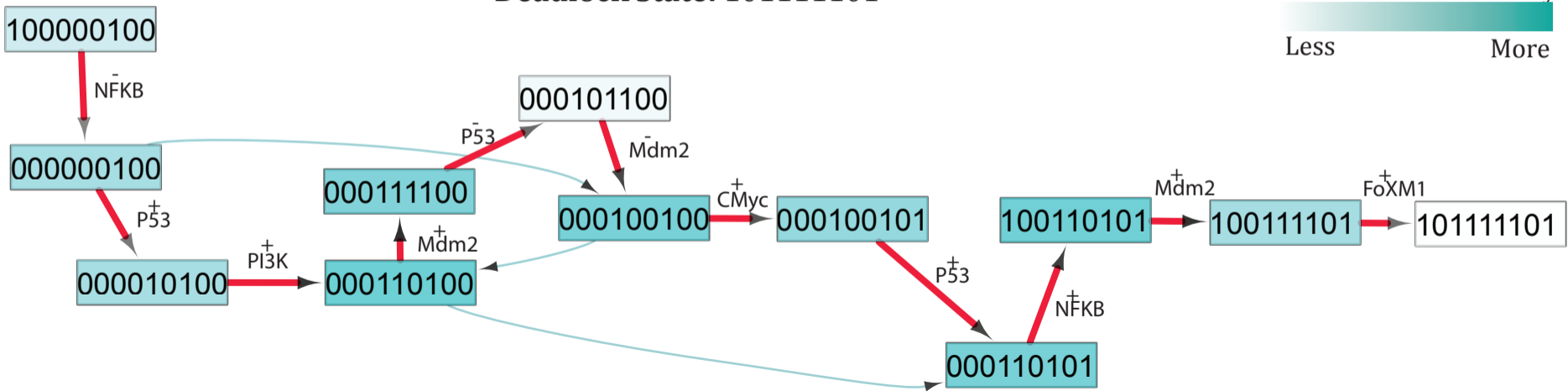

Supplement: Supplemental Information 9 — Each node in the graph represents a unique state of the system characterized by qualitative expression of genes in the following order: (NF-κB, p21, FoxM1, PI3K, p53, Mdm2, OGT, OGA, c-Myc). Activation of a particular gene/entity is indicated as “1”, whereas “0” indicates and expression level for the particular gene that is below the activation threshold. Nodes are shaded based on the level of betweenness centrality. Each transition is labelled with change in expression level of gene (”+” shows increase and ”-” shows decrease). Nodes and transitions typically associated with tumor progression are represented with arrows colored in red. The nodes and trajectories involved in recovery are represented with arrows colored in green. The trajectories start at state “100000100” and finally lead to a deadlock state (“101111101”). [file peerj-04-2348-s009.pdf]

**Deadlock State: 10111101**

More

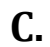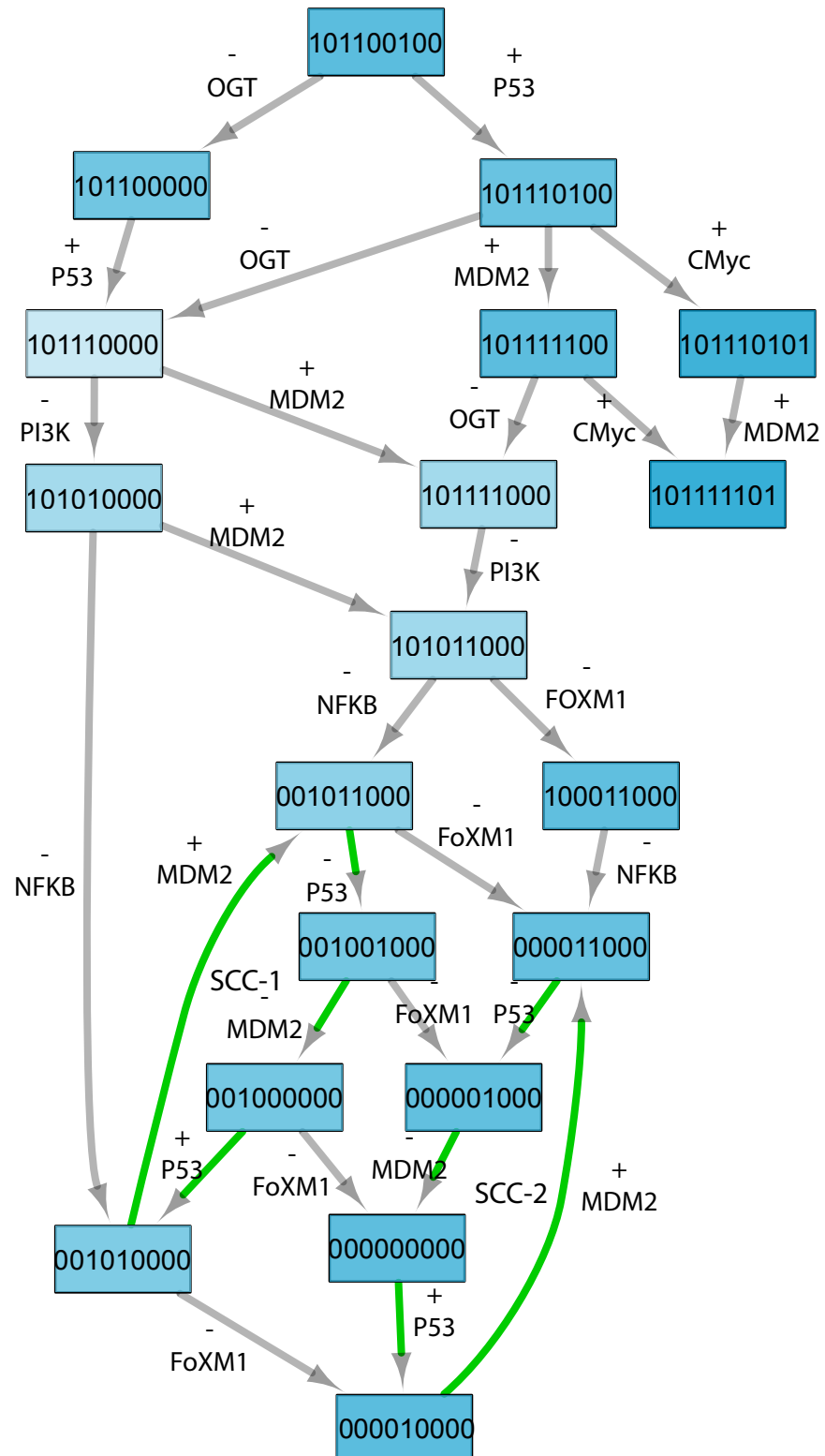

Supplement: Supplemental Information 10 — Trajectories from start state (1000000100) to recovery state (shown in A). Trajectories from advanced tumor progression to recovery state (000000000) (B). Transition from recovery state to attractor (C).The subgraph of the state transition graph highlights possible recovery from advanced tumor progression. Each node in the graph represents a unique state of the system characterized by the expression level of critical genes (NF-κB, p21, FoxM1, PI3K, p53, Mdm2, OGT, OGA, c-Myc). Activation of a particular gene/entity is indicated as “1”, whereas “0” indicates and expression level for the particular gene that is below the activation threshold. Nodes are shaded based on the level of betweenness centrality. Each transition is labelled with change in expression level of gene (”+” shows increase, ”-” shows decrease). The trajectories involved in oscillations are represented with arrows colored in green. The trajectories start at state “101100100” and finally lead to either recovery (“000000000”) or a deadlock state (“101111101”). [file peerj-04-2348-s010.pdf]
