# Supplementary material for: Formal modeling and analysis of the hexosamine biosynthetic pathway: role of O-linked N-acetylglucosamine transferase in oncogenesis and cancer progression
Source: PeerJ. 2016 Sep 27;4:e2348. doi: 10.7717/peerj.2348 (PMC5047222; doi:10.7717/peerj.2348)
Supplement: Supplemental Information 11 [file peerj-04-2348-s011.pdf]

Table 1: The software/Tools used in this study (along with the Download links)

Method	Tools	Link
Qualitative Modeling	GinSim	http://ginsim.org/
	GenoTech	http://mac.softpedia.com/get/Math-Scientific/Genotech.shtml
Parameter Estimation	SMBioNet	http://www.i3s.unice.fr/∼richard/smbionet/
Network Analysis	Cytoscpae	http://www.cytoscape.org/
Hybrid Modeling	HyTECH	https://embedded.eecs.berkeley.edu/research/hytech/
